# Prevalence and risk factors of tinea capitis in primary school children across four regions of Cameroon

**DOI:** 10.1016/j.nmni.2025.101667

**Published:** 2025-11-13

**Authors:** D.A.J. Agokeng, G.S.S. Njateng, S. Dabou, K. Diongue, K.B.D. Agokeng, S. Ranque

**Affiliations:** a IHU Méditerranée Infection, 13385, Marseille, France; b Aix Marseille Université, Assistance Publique-Hôpitaux de Marseille, Service de Santé des Armées, RITMES, 13005, Marseille, France; c Service de Parasitologie-Mycologie, Faculté de Médecine, de Pharmacie et D’Odonto-stomatologie, Université Cheikh Anta Diop de Dakar, BP 16477, Dakar, Senegal; d Laboratoire de Parasitologie et de Mycologie, Hôpital Aristide Le Dantec, BP 5005, Dakar, Senegal; e Research Unit of Biochemistry of Medicinal Plants, Food Sciences and Nutrition, Department of Biochemistry, Faculty of Science, University of Dschang, P. O Box 67, Dschang, Cameroon; f Research Unit of Microbiology and Antimicrobial Substances, Department of Biochemistry, Faculty of Science, University of Dschang, P. O Box 67, Dschang, Cameroon

**Keywords:** Epidemiology, Dermatophytes, Dermatophytosis, *Tinea capitis*, Schoolchildren, *Microsporum audouinii*, *Trichophyton rubrum*, *Trichophyton soudanense*

## Abstract

Tinea capitis (TC), commonly known as scalp ringworm, is a dermatophytosis affecting the scalp. It represents a significant public health concern worldwide, particularly in Africa. However, epidemiological data on this infection remain limited in Cameroon. This study aimed to assess the prevalence and risk factors of TC among school-children in four regions of Cameroun. A cross-sectional study was carried out from April to June 2023 including pupils aged 5–14. First, a standardized questionnaire was administered to the participants to collect sociodemographic data. Then, the children were examined and TC lesion samples were collected and cultured onto Sabouraud Chloramphenicol-Gentamicin Agar. The cultured dermatophytes were identified based on their morphological features and with MALDI-TOF mass spectrometry.

A total of 459 children were included, of whom 118 (25.7 %) presented with TC lesions. Traditional hair braiding (OR = 0.24, 95 %CI: 0.06–0.90), and sleeping alone (OR = 0.49, 95 %CI: 0.26–0.91) were associated with a decreased risk of TC in multivariate logistic regression analysis. In contrast, male sex (OR = 3.15, 95 %CI [1.63–6.06]), hairdressing at home (OR = 2.39, 95 %CI [1.45–3.93]), and ringworm in siblings (OR = 2.79, 95 %CI [1.73–4.50]) were associated with an increased risk of TC. These results emphasise the importance of raising awareness and providing education on hygiene and hairstyling practices. Further efforts are needed strengthen health infrastructure, and implement targeted public health programmes to better control this disease in Cameroon.

## 1 Introduction

Superficial fungal skin infections, also referred to as dermatophytosis, are among the most common fungal diseases worldwide, causing chronic morbidity, particularly among children in developing countries [1]. They are caused by a group of related filamentous fungi known as dermatophytes [2,3]. Dermatophytes are classified as anthropophilic, zoophilic and geophilic dermatophytes depending on whether they are transmitted from one infected human to another, acquired through contact with infected animals or contracted from contaminated soil or fomites, respectively [4]. The main genera include *Trichophyton, Epidermophyton* and *Microsporum* [5]). They are distributed worldwide, with most infections reported in Africa, Asia, Southern and Eastern Europe [3,6]. Tinea capitis (TC), the most common dermatophytosis in children [7], affects the scalp and may cause hair breakage, sometimes associated with inflammation or permanent alopecia [8]. Several factors are associated with the spread of the disease, including geographical setting, healthcare access, migration, overcrowding, environmental sanitation, personal hygiene, socioeconomic status, and age [9,10]. Likewise, TC is most frequent in tropical areas, where high temperature and humidity favour increased incidence [11]. Climatic conditions and seasonal variations directly influence the incidence of certain dermatophytoses [12]. The frequency of TC varies according country, with a continual epidemiological evolution [13]. Although a few studies have been conducted in Cameroon [14,15], epidemiological data on TC are still lacking across most of the national territory. Therefore, to address this gap, the present study aimed to assess the epidemiological profile of TC among schoolchildren in four regions of Cameroon.

## 2 Patients and methods

### 2.1 Study sites

The main reason for choosing the city of Maroua for this study is that it is a cosmopolitan city comprising several social groups engaged in agriculture, livestock, trade, and crafts. Similar to the rest of the Far North region, Maroua has a Sudano-Sahelian climate marked by a dry season from October to May and a short rainy season from June to September. The average annual rainfall is about 700 mm. The thermal differences are significant, with extreme temperatures ranging from 27 to 41 °C. Temperatures are generally lower during the rainy season and at night in the dry season. The relative humidity of the air is quite low and varies with altitude. It varies between 30 and 35 %, whereas the potential evaporation is considerable, from 3500 to 3700 mm [16].

Garoua, the capital of the North Region of Cameroon, is a river port city. It is located at 9.30° N latitude and 13.40° E longitude. It has a tropical Sudanian climate with a dry season (October to April) and a shorter rainy season (May to September). The average annual rainfall amounts to 1000 mm. Temperatures remain high with an average of 28 °C and maximum temperatures of 40–45 °C in March and April. However, large irregularities can be observed [17].

The Adamaoua region covers an area of 63,701 km^2^ with a population of about 1,080,500 inhabitants, corresponding to a density of 17 inhabitants per km^2^. It is located between the 6th and 8th degrees of North latitude, the 11th and 15th degrees of East longitude. The climate is Sudanian tropical, and characterised by two seasons: the dry period goes from November to April then comes the wet season. Temperatures are between 22 °C and 25 °C while annual averages of rainfall are 900 mm–1500 mm and decrease further to the north. The predominant vegetation is tree savannah. There are also numerous forests along the humid valleys. The soil is ferralitic and volcanic. The main economic activities are agriculture, livestock, fishing and beekeeping [18].

Bertoua, a city of over 200,000 inhabitants, is located in the equatorial forest zone and is crossed by several river systems including the Nyong, Dja, Lom, Kadéï, Boumba, and Ngoko. The region is part of the Sahelo-Sudanese domain, characterised by four seasons including two rainy seasons (a short one from September to November and a long one from March to June) and two dry seasons (from November to March and from July to August for the long and the short, respectively) [19].

### 2.2 Population and sample collection

**Fig. 1:**
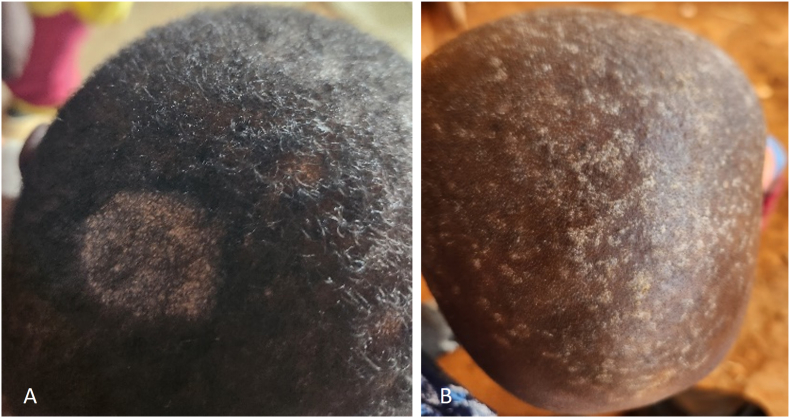
Tinea capitis clinical presentation: the microsporic form is characterised by the presence of few large alopecia patches (A) and the trichophytic form is characterised by the presence of numerous small alopecia patches (B). Prevalence of TC in each study site.

The study was carried out during the rainy season, from April to June, in eight primary schools of the four regions. Using randomisation, one primary school in a rural area and another in an urban area were selected in each region (Fig. 1). It comprises the public schools of Djarengol and Lowol Diga for the Far North Region, Bocklé and Kolléré for the North Region, Burkina and Ta-Ifa for the Adamaoua Region, and Tigaza and Nkolbikon for the East Region. Pupils, aged 5–13 years, were selected in each primary school using a block randomisation design adjusted on the number of pupils in each classroom. First, parents or guardians provided written informed consent on behalf of the participating children. The sociodemographic data were collected using a standardized questionnaire. Schoolchildren absent during the study period, as well as those receiving antifungal treatment or who had taken antifungals within 14 days before sampling, were excluded in the study. After clinical examination, skin areas showing signs compatible with dermatophytosis were sampled using a sterile compress. The samples were stored at room temperature and, less than one month after sampling, they were sent, in agree with the WHO recommendations on the international transport of patient samples [20], to the Parasitology and Mycology laboratory at the University Hospital of Marseille, France, for analysis.

### 2.3 Laboratory procedures

Each specimen was inoculated on Sabouraud dextrose agar (SDA) plates supplemented with chloramphenicol and cycloheximide (*Thermo Scientific*™, Germany) and then incubated at 27 °C up to four weeks. Identification of dermatophyte species was based on macro-morphological examination of colonies, further using Matrix Assisted Laser Desorption Ionization - Time of Flight Mass Spectrometry (MALDI-TOF MS), and rDNA internal transcribed spacer (ITS) sequence analysis as previously described [21,22].

### 2.4 Statistical analysis

The data were analysed using the IBM SPSS version 20.0 for Windows software. Continuous variables were expressed as mean with the standard deviation (SD), while categorical variables were expressed as percentages with the 95 % confidence intervals (CI). Continuous variables were compared using the ANOVA test. Categorical variables were compared using the Chi-square test or Fisher's exact test, where appropriate. All statistical tests were two-sided with a *p* < 0.05 significance level threshold. Univariate and multivariate logistic regression analyses were performed to estimate odds ratios (ORs) with 95 % CIs. All covariates with a *p* < 0.20 significance level in the univariate analysis were included in the multivariate logistic regression model. A stepwise selection was performed to retain the most parsimonious model including the covariates that displayed an independent statistically significant (*p* < 0.05) effect on tinea capitis infection risk.

### 2.5 Ethical considerations

The study protocol was reviewed and approved by the Institutional Review Board of the Faculty of Medicine, University of Douala, Cameroon (N^o^3146 CIE-Udo/06/2022/T). The study protocol was also approved by Local Education Authorities at each study site. Parents or guardians provided written informed consent on behalf of the participating children.

## 3 Results

### 3.1 Characteristics of the study population

A total of 459 children were included and clinically examined in this study. The mean age of the participants was 9.5 ± 2.5 years (range: 5–14 years). There were 289 (63.0 %) males, and 230 (50.1 %) lived in urban areas. Overall, biological samples were collected from 118 (25.7 %) children who presented with lesions compatible with TC (Fig. 1).

**Fig. 2:**
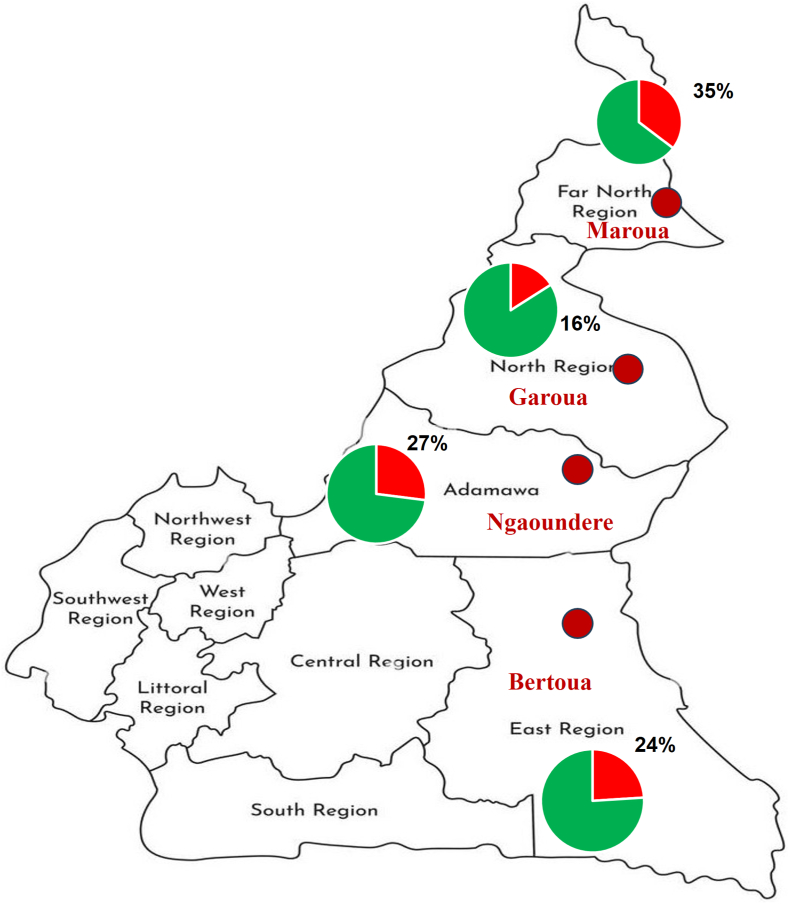
Map of Cameroon showing the location and the prevalence of tinea capitis in each of the study sites. *(The base map was downloaded from the website:*https://www.alamyimages.fr/illustration-isolee-vectorielle-de-la-carte-administrative-simplifiee-du-cameroun-frontieres-et-noms-des-regions-silhouettes-de-lignes-noires-image566442398.html?imageid=AC83F412-65FF-407D-BDD9-599C3CAB65CE%26pn=1%26searchId=b7246f52aee98f24a7547d5c11e2326b%26searchtype=9.

Of the 459 children, 118 presented with lesions compatible with TC which represents an overall prevalence of 25.7 %. The prevalences in each study site are detailed in Fig. 2.

### 3.2 Factors associated with TC

Factors associated with TC in the univariate analysis are presented in Table 1. There was no statistically significant association between TC and hair salon attendance (*p* = 0.748), the presence of enclosure close to the house (*p* = 0.896), as well as the garbage depot near the house (*p* = 0.321). In contrast, TC was significantly more frequent in children sleeping alone (*p* = 0.048), in children with regular shaving of the head (*p* = 0.001), those who have hairdressing at home (*p* = 0.003, children with ringworm in the siblings (*p* =<0.0001), and those making traditional braiding hair (*p* = 0.002).

**Table 1:** Univariate analysis of tinea capitis risk factors among school children in Cameroon.

Factors	Total Population N = 459 (%)	Tinea capitis N = 118 (%)	No tinea capitis N = 341 (%)	*p* value
Settlement				
Rural area	229 (49.9)	68 (57.6)	161 (47.2)	0.051
Urban area	230 (50.1)	50 (42.4)	180 (52.8)	
**Sex**				
Male	289 (63)	93 (78.8)	196 (57.5)	<0.0001
Female	170 (37)	25 (21.2)	145 (42.5)	
**Hairdressing habit**				
Hairdressing at home	274 (60)	84 (71.2)	190 (55.7)	0.003
Hair salon attendance	181 (40)	48 (40.7)	133 (39)	0.748
Regular shaving of the head	279 (60.8)	87 (73.7)	192 (56.3)	0.001
Sleep alone	91 (19.8)	16 (13.5)	75 (22)	0.048
**Others**				
Tinea capitis in siblings	124 (27)	50 (42.4)	74 (21.7)	<0.0001
Presence of enclosure close to the house	231 (50.3)	60 (50.8)	171 (50.1)	0.896
Garbage depot near the house	227 (49.5)	63 (53.4)	164 (48)	0.321
Traditional braiding hair	135 (29.4)	14 (11.9)	121 (35.5)	0.002

Factors such as age, rural residence, traditional hair braiding, and sleeping alone were associated with a decreased risk of TC. In contrast, male sex, hairdressing at home, regular head shaving, and the presence of ringworm in siblings were associated with an increased risk of TC in children **(**Table 2**)**.

**Table 2:** Univariate and multivariate unconditional logistic regression analyses of the tinea capitis risk factors identified in this population.

Factors	Univariate analysis			Multivariate analysis		
	OR	IC 95 %	*p* value	OR	IC 95 %	*p* value
Sex						
Age	0.91	[0.84–1]	0.037	0.93	[0.85–1.03]	0.151
Male	2.75	[1.68–4.5]	<0.0001	3.15	[1.64–6.06]	0.001
**Settlement**						
Rural area	1.52	[1–2.32]	0.052	0.75	[0.5–1.2]	0.204
Hairdressing at home	1.96	[1.25–3.09]	0.003	2.4	[1.45–3.94]	0.001
Traditional braiding hair	0.26	[0.11–0.65]	0.004	0.24	[0.06–0.9]	0.035
Regular shaving of the head	2.18	[1.37–3.46]	0.001	1.10	[0.6–2.04]	0.755
**Hairdressing habit**						
Sleep alone	0.56	[0.31–1]	0.050	0.49	[0.3–0.9]	0.025
Ringworm in siblings	2.65	[1.7–4.15]	<0.0001	2.8	[1.7–4.5]	<0.0001
Hair salon attendance	1.07	[0.7–1.64]	0.748	–	–	–
**Others**						
Close contact with domestic animals	1.28	[0.82–1.97]	0.272	–	–	–
Presence of enclosure close to the house	1.03	[0.7–1.563]	0.896	–	–	–
Garbage depot near the house	1.24	[0.81–1.88]	0.322	–	–	–

Among the 28 cultured dermatophytes, *T. soudanense* was the most frequently isolated species (57.1 %), followed by T. rubrum (32.1 %), T. violaceum (7.1 %), and M. audouinii (3.6 %).

## 4 Discussion

This study aimed to assess the epidemiological profile of TC among schoolchildren across four regions of Cameroon. Many studies reported TC as an important clinical problem widely distributed worldwide, especially among children [23]. The main finding was an overall prevalence of 25.7 % for TC among schoolchildren. This prevalence is almost comparable to the 21.1 % reported by Mirata et al. (2025) in a review of studies conducted in Ethiopia [24]. This prevalence was higher than that reported in other African settings, including Meiganga, Cameroon (8.1 %) [14] and Mauritania (10.5 %) [25]. This demonstrates that TCs are still endemic in schoolchildren in Cameroon. The prevalence of TC was higher in rural areas (57.6 %) compared to urban areas (42.4 %). This could be due to poor sanitation in rural areas and more frequent contact with domestic animals [25]. Likewise, communal life is more profound among the majority of rural inhabitants, resulting in the exchange of personal effects such as clothing that could serve as vehicles of transmission of superficial fungal infections [26]. **Ayanbimpe *et al*** [27] in Nigeria and **Coulibaly *et al*** [28] in Mali obtained different results with 45 % in urban areas and 23 % in rural areas in some parts of central Nigeria; 55 % in urban areas and 17 % in rural areas in Mali, respectively. This study found a significant association between male sex and TC, with 78 % of cases occurring in boys, consistent with previous reports. The same trends were obtained by Ref. [6] in Kenya, who found that males (45.3 %) were most infected compared to females (36.7 %). This can be explained by boys' shorter hair (easier contamination by spores) and by the greater contact compared to girls with domestic or stray animals, which are often asymptomatic carriers [29]. Home hairdressing was associated with a significantly higher prevalence (71.2 %), and regular head shaving with 73.7 %, compared to those without these practices. **Pérez-Tanoira *et al****.* [30] notes that informal hairdressing practices, common areas, may lack proper hygiene, increasing infection risk and that shaving can cause minor scalp injuries, providing entry points for fungal pathogens. The presence of TC in siblings was a highly significant risk factor, with a prevalence of 42.4 %. Multivariate analysis showed, in particular, that it increases the risk of developing scalp ringworm by 2.8 times. This increased TC risk might be due to the promiscuity between family members, as well as the sharing of several fomites, including combs and towels. A similar observation has been reported in Ethiopia by **Hibstu *et al.*,** [24,31]. In contrast, **Koudoukpo *et al.*** [32] in a study in Benin showed that the existence of TC in the entourage was not significantly associated with the occurrence of TC.

One limitation of this study was the relatively high rate of false-negative dermatophyte cultures, resulting in a limited number of positive isolates. In contrast to **Agokeng *et al*** [15], who reported a higher prevalence of *M. audouinii* (43.4 %) in Dschang, Cameroon, this study identified *T. soudanense* as the most prevalent species (57.1 %). This is consistent with the fact that *T. soudanense* is one of the most common dermatophyte species in Central Africa [33].

## 5 Conclusions

This study revealed a considerable prevalence of TC among school-aged children in four regions of Cameroon, with an overall prevalence of 25.7 %. The findings suggest that male sex, home hairstyling, and having siblings with TC increase the risk of infection. Conversely, practices such as traditional braiding and sleeping alone appear to reduce this risk. These findings highlight the need for awareness and educational programmes on hygiene and hairstyling practices, particularly in rural areas where poor living conditions facilitate the transmission of dermatophytoses. Further efforts are required to expand epidemiological studies to other regions, strengthen health infrastructure, and implement public health interventions to better control TC in Cameroon.

## CRediT authorship contribution statement

**D.A.J. Agokeng:** Writing – original draft, Methodology, Investigation, Formal analysis, Data curation, Conceptualization. **G.S.S. Njateng:** Writing – review & editing, Validation, Supervision, Conceptualization. **S. Dabou:** Writing – review & editing, Validation, Methodology, Formal analysis, Data curation. **K. Diongue:** Writing – review & editing, Validation. **K.B.D. Agokeng:** Writing – review & editing, Investigation. **S. Ranque:** Writing – review & editing, Validation, Supervision, Resources, Methodology, Conceptualization.

## Funding

This study was funded by the 10.13039/100019714Foundation Méditerranée Infection.

This work was supported by a grant from the French Government managed by the National Research Agency under the “Investissements d'avenir (Investments for the Future)” programme with the reference ANR-10-IAHU-03 (Méditerranée Infection), by the Contrat Plan Etat-Région and the European funding 10.13039/501100008530FEDER IHUPERF.

## Declaration of competing interest

The authors declare the following financial interests/personal relationships which may be considered as potential competing interests:Armel AGOKENG reports financial support was provided by Fondation Méditerranée Infection. Reports a relationship with that includes:. Has patent pending to. If there are other authors, they declare that they have no known competing financial interests or personal relationships that could have appeared to influence the work reported in this paper.
